# Spatial Dynamics and Expanded Vertical Niche of Blue Sharks in Oceanographic Fronts Reveal Habitat Targets for Conservation

**DOI:** 10.1371/journal.pone.0032374

**Published:** 2012-02-29

**Authors:** Nuno Queiroz, Nicolas E. Humphries, Leslie R. Noble, António M. Santos, David W. Sims

**Affiliations:** 1 Marine Biological Association of the United Kingdom, The Laboratory, Citadel Hill, Plymouth, United Kingdom; 2 CIBIO – U.P., Centro de Investigação em Biodiversidade e Recursos Genéticos, Campus Agrário de Vairão, Rua Padre Armando Quintas, Vairão, Portugal; 3 School of Biological Sciences, University of Aberdeen, Aberdeen, United Kingdom; 4 School of Marine Science and Engineering, Marine Institute, University of Plymouth, Plymouth, United Kingdom; 5 Ocean and Earth Science, National Oceanography Centre, University of Southampton, Waterfront Campus, Southampton, United Kingdom; 6 Centre for Biological Sciences, University of Southampton, Highfield Campus, Southampton, United Kingdom; Institut Pluridisciplinaire Hubert Curien, France

## Abstract

Dramatic population declines among species of pelagic shark as a result of overfishing have been reported, with some species now at a fraction of their historical biomass. Advanced telemetry techniques enable tracking of spatial dynamics and behaviour, providing fundamental information on habitat preferences of threatened species to aid conservation. We tracked movements of the highest pelagic fisheries by-catch species, the blue shark Prionace glauca, in the North-east Atlantic using pop-off satellite-linked archival tags to determine the degree of space use linked to habitat and to examine vertical niche. Overall, blue sharks moved south-west of tagging sites (English Channel; southern Portugal), exhibiting pronounced site fidelity correlated with localized productive frontal areas, with estimated space-use patterns being significantly different from that of random walks. Tracked female sharks displayed behavioural variability in diel depth preferences, both within and between individuals. Diel depth use ranged from normal DVM (nDVM; dawn descent, dusk ascent), to reverse DVM (rDVM; dawn ascent, dusk descent), to behavioural patterns where no diel differences were apparent. Results showed that blue sharks occupy some of the most productive marine zones for extended periods and structure diel activity patterns across multiple spatio-temporal scales in response to particular habitat types. In so doing, sharks occupied an extraordinarily broad vertical depth range for their size (1.0–2.0 m fork length), from the surface into the bathypelagic realm (max. dive depth, 1160 m). The space-use patterns of blue sharks indicated they spend much of the time in areas where pelagic longlining activities are often highest, and in depth zones where these fisheries particularly target other species, which could account for the rapid declines recently reported for blue sharks in many parts of the world's oceans. Our results provide habitat targets for blue shark conservation that may also be relevant to other pelagic species.

## Introduction

Information on movements and behaviour is an often overlooked, but nevertheless crucial part of assessing population trends of mobile animals since at specific locations movement greatly influences temporal changes in population density [1]. However, for many exploited marine animals such as large fishes, accurate fisheries-independent population assessments of space use are still lacking, hampering effective conservation and management efforts. Understanding patterns of habitat use, both horizontal and vertical, in marine predators with regards to physical features of the environment is important because they influence predators' movements and distribution to a large degree [2], and aid our prediction of animal behaviour in the face of changing environmental conditions [3]. Moreover, such information is valuable for management purposes, especially in species that are at risk from expanding fisheries [4], [5]. In the North Atlantic alone, many pelagic species which undergo long-distance movements or migrations are vulnerable to large-scale fishing pressure, either from directed or incidental captures [5], [6], [7]. In particular, the estimated abundance of oceanic sharks has declined by between 21 and 99% in recent years when compared with estimates preceding extensive exploitation [8], [9]. Commonly reported as the most frequently caught shark species [e.g. 10], [11], the blue shark *Prionace glauca* is no exception, with estimated declines in some regions of 60–80% since the 1980s and 1990s [6], [12].

Longline surveys and mark-recapture studies in the Atlantic have provided considerable information on the distribution and extensive movements of pelagic sharks, and for blue sharks in particular [13]. These studies naturally fail, however, to provide a detailed understanding of how pelagic sharks respond to dynamic changes in ocean habitat type. Previous studies on plankton-feeding pelagic sharks have quantified the importance of productive areas such as oceanographic fronts (boundaries between different water mass types) for foraging opportunities [14], [15], [16] or by highlighting the behavioural tactics such species use when faced with habitat changes [17]. A similar understanding for horizontal movements of predatory pelagic sharks, that are often highly migratory and that can move long distances over relatively short time periods, is not so well established. In the Pacific Ocean there is quantitative evidence for area-restricted movements by predatory sharks [18], [19] characteristic of foraging occurring in productive regions, and it is reasonably well documented in tunas [3], [20]. However, no comparable studies on predatory pelagic sharks have been undertaken in the Atlantic Ocean, where longline fishing pressure is up to eight-fold higher (Fig. S1). In this context, it is important to determine association rates of Atlantic sharks with strong environmental gradients (e.g. sea surface temperature, SST) that are often targets for intensive fishing activities [21], [22].

Understanding the patterns of vertical movement in relation to environmental variations will identify the locations and depths occupied by sharks, underpinning much-needed assessments of overlap with depth ranges targeted by longline fisheries in the region. Vertical movements, in particular diel depth changes, have been recorded in diverse marine species, from zooplankton to large vertebrates [e.g. 17], [23], [24]. Diel vertical migration (DVM) in a wide range of zooplankton species is thought to be a trade-off between reduced predation risk with increasing depth, and improved feeding opportunities in prey-rich surface waters [23], [25], [26]. It is generally accepted that relative changes in light intensity are also a principal driver of diel migrations [27]. Hence, so-called normal DVM is often characterised by an ascent into shallow water at sunset followed by a descent at sunrise to greater depths (dusk – ascent; dawn – descent). In addition, diel migration patterns may be regulated by physical oceanographic structures (stratification, eddies) and by the depth of the chlorophyll maximum layer [28]. Similar changes in the timing of vertical migration have been observed in larger invertebrates [e.g. jellyfish], [ squid; 29,30] and also small fish [31]. Therefore, it is not surprising that large predators, such as sharks and tunas, modify their diving behaviour in response to diel migrating prey. As a result, diel patterns of activity in apex predators have frequently been linked to foraging or search behaviour [32], [33]. In these studies pelagic fish displayed consistent diel patterns of vertical movement at different temporal scales, geographic regions and across life-history stages (i.e. juveniles, adults), frequently with increased diving activity rhythms at specific periods of the day. Additional theories to explain diel changes in behaviour have also been proposed. For example, diel depth changes in a demersal shark have been linked to increased bioenergetic efficiency, whereby sharks hunt in warm surface waters at night and rest in cooler waters during the day [34]. It has also been shown that fish may alter diving behaviour in an attempt to minimise predation risk [35].

In this study, pop-off satellite-linked archival transmitter (PSAT) tags were deployed on blue sharks at two different latitudes with varying oceanographic features, including frontal zones, to determine patterns of space use and their linkage with physical characteristics of the environment. We also examined diel vertical behaviour in relation to oceanographic features to investigate whether behavioural patterns were related to temperature (thermal structure) or probable prey movements.

## Methods

### Study species

The blue shark is a wide-ranging shark occurring in all tropical and temperate seas. Distribution of the Atlantic population is complex with spatial and temporal segregation by sex and age, in addition to short and longer range seasonal and annual migrations, including trans-Atlantic and trans-equatorial movements [36]. In the eastern Atlantic adult females are found around the Canary Islands and North Africa in winter, many of which are pregnant [36]. Adult males are found further north, mainly off Portugal, along with juveniles and sub-adult females, the latter group undertaking a summer migration into the western English Channel and Irish waters [37], [38]. Adult males and juveniles are also found in offshore regions, particularly off the Azores [39]. Mark-recapture studies have, however, also shown apparent long-term site fidelity to specific, relatively localised regions by some components of the population [40], although such studies are largely influenced by the spatial and temporal distribution of fishing effort, and likely reflect the movement of the fishing vessels rather than describing actual movement or residence patterns of blue sharks [41].

### Shark tagging

**Figure 1:**
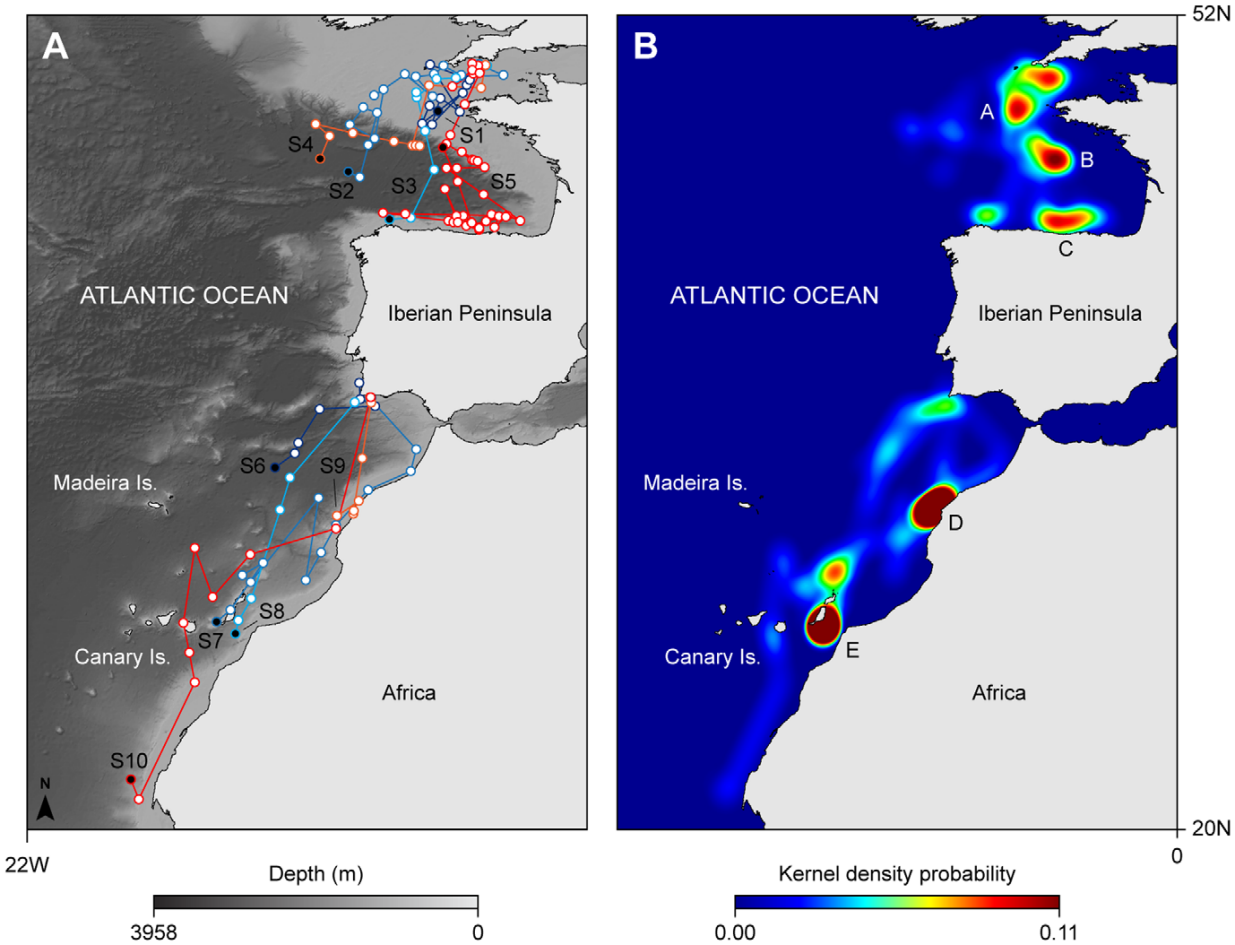
Movement and high space use areas occupied by PSAT-tagged blue sharks. (A) General movement patterns overlaid on bathymetry; black circles denote pop-up locations and white circles the geolocated positions. (B) Kernel density plot showing five major areas of prolonged residency labelled A–E.

Blue sharks were captured using rod and line and brought onboard for body-length measurement and tagging. Fishing took place between July 2006 and June 2008 in two areas: the English Channel off south-west England and off southern Portugal (Fig. 1A). A total of 16 blue sharks were tagged with PSAT tags in these two sites. Shark 1 was tagged with a Mark 4 PSAT tag (PAT4, Wildlife Computers, WA, USA), which records depth (maximum: 1000 m; accuracy: 0.5 m), water temperature (range: −40 to 60°C; accuracy: 0.05°C) and light level (at 550 nm wavelength) and relays data via an Argos-certified satellite transmitter. The tag was programmed to sample each parameter every 10 s and detach after 30 days. The remaining sharks (#2–16) were tagged with Mk10 PSAT tags (Wildlife Computers). Parameters were sampled throughout the deployment at varying intervals (from 1 to 10 s) and stored as summary data over set intervals of 4 or 6 h. For each integration interval, PSAT tags relayed information on the minimum and maximum depth obtained and selected temperatures across this range so that temperature/depth profiles could be generated. Tags were programmed to detach after 60, 90, 120 or 180 days after tagging. In 2006/2007, PSATs were attached via a 20-cm long monofilament tether (250 lb test) tether connected to a 5 cm long stainless steel T-bar arrowhead; in 2008 tags were rigged with a 15-cm monofilament tether and an ‘umbrella’ type nylon dart. All tags were inserted into the dorsal musculature at a 45° angle to a maximum depth of 10 cm.

### Horizontal movements and space-use

The movement of each shark was estimated using either satellite relayed data from each tag or from archival data after the tags were physically recovered. Positions of each shark between attachment and tag pop-up were reconstructed using software provided by the manufacturer (WC-GPE, global position estimator program suite), where daily maximal rate-of-change in light intensity is used to estimate local time of midnight or midday for longitude calculations, and day-length estimation for determining latitude. Anomalous longitude estimates resulting from dive-induced shifts in the estimated timings of dawn and dusk from light curves were discarded from the dataset. Geolocations >3° of longitude from the previous longitude estimate were also removed [16]. Latitude along the longitude was then corrected by matching minimum and maximum tag-recorded water temperatures from the shallowest bin recorded in each period to sea surface temperature (SST) values on night-time, 8-day composite, moderate-resolution, imaging spectroradiometer (MODIS) remote-sensing images. The most parsimonious location was derived by matching individual pixel SSTs within a variable-sized circular area (radius 100–500 km) around the previous tag position. If no matches were obtained within the smaller area, the radius was increased until matches occurred. Variable-sized areas were used because geolocations at constant intervals were not always determined due to gaps in the data. A geographic mean position was calculated from all possible pixel locations within this area to derive each shark position. Maximum dive depth was compared with seabed depths from a digital bathymetry map (general bathymetric chart of the oceans GEBCO 30″ dataset) within each area to filter anomalous positions where the dive depth was greater than seabed depth. Using the ArcGIS geographical information system (ESRI Inc., CA, USA), intermediate waypoints were applied to track steps where trajectories between located positions crossed land. The final estimated positions were then analysed point-to-point with a 1 m s^−1^ swim speed filter, which is the maximum over-the-ground swimming speed measured for blue sharks [42] and is consistent with swimming speeds of other pelagic sharks. A position separated from an adjacent position by a distance too great to achieve in the speed-filter-imposed time between those points was shifted to a location along the track where the forced speed limit provided an acceptable distance. Previous studies using this general method estimated the mean error distance of light/SST geolocation compared to tagging or pop-up locations to be 75.5 km±54.5 S.D. (range: 36.9–183.9 km) [16] and 78.0 km±21.4 S.D (range: 54.7–100.0 km) [43].

Shark tracks were initially partitioned into 1 h data points; the interpolated points were then plotted against a 0.5×0.5° grid in ArcGIS. Total time spent in each grid cell was calculated by summing the number of hourly points located within them (Fig. S2A). To test if the observed movement patterns (and in particular the aggregations at some cells) were different from simple random movement, a Brownian normal diffusion random-walk model was used. For each simulation, 10 particles (model sharks) were started from points corresponding to the actual tagging positions of blue sharks (five were released off southwest England and five off southern Portugal). The movement path of each particle comprised a sequence of discrete steps and turning angles, with the former limited by the actual number of individual steps recorded for each tracked shark. In each iteration, step length was drawn from a normal distribution with an average and standard deviation estimated from tracked sharks and limited by the minimum and maximum step lengths observed. Angles were derived from a uniform distribution between −180° and +180°. After computing a new position, a check was made to ensure that it did not fall within land masses. If so, the position was rejected and a new angle and step length were drawn. At the end of each simulation, a final map with a 0.5×0.5° grid was constructed with the total number of hours computed for each grid cell (for examples see Fig. S2B–D). A total of 5000 simulation runs of 10 model sharks were completed. To test whether the observed pattern differed significantly from the random-walk generated patterns, the distribution of the mean/variance ratio of the number of hours per cell was computed using the simulations. The mean/variance proportion was also computed for the observed data and was compared with the distribution computed previously to estimate its *p* value [44].

Interpolated data points were also used to calculate shark space use by performing a kernel density estimate in ArcGIS [spatial analyst/kernel density]. Since data was not normally distributed (Shapiro-Wilk; *W* = 0.733, *p*<0.001), a Spearman Rank correlation was carried out to test whether there was a significant relationship between the estimated space use and enhanced primary production (using mean chlorophyll *a* concentration as a proxy). Similarly, hourly interpolated vessel monitoring system (VMS) data from surface longliners (tracked between 2006 and 2008 in the summer/autumn period) were used to estimate kernel point density; a Spearman Rank correlation was subsequently performed between the estimated longliner space use patterns (data not normally distributed; Shapiro-Wilk; *W* = 0.863, *p*<0.001) and mean chlorophyll *a* levels.

### Vertical movement analysis

A modified version of the split-moving window (SMW) method was employed to detect significant shifts in time-at-depth (TAD) data and, thus, define behavioural phases [3], [45]. Briefly, a variable-sized split window ranging from 4 or 6 h to a maximum of 5 days was used to compute dissimilarities between the two halves of the window along the time steps of the vertical track. A multivariate measure of dissimilarity (Euclidean) was computed between every possible pair of samples from different halves and the values of all comparisons were then averaged. The value obtained was assigned to the centre of the window, which then moved one step forward, repeating this process until the window reached the end of the data series. Statistical significance of dissimilarities for each window's midpoint was computed using a randomisation procedure. The result from each window size was then plotted by piling them vertically, resulting in an inverted triangle with the lower vertex pointing to the boundary location whenever a significant shift was detected [3], [45]. This modified method has the advantage of not requiring evenly distributed data, which is suitable for satellite-transmitted summary data, given that it often has data gaps due to the limited bandwidth of the Argos satellite relay system. This limits data recovery rates during the data upload period when the tag is at the sea surface and prior to its batteries becoming exhausted (∼14 days). Data for each behavioural phase was pooled and summarized as diel frequencies of time spent at depth, to distinguish between different patterns of behaviour that could be similar among sharks. T-tests were used to determine whether there were significant day/night differences in maximum depth and were performed at the *p* = 0.05 level of significance. To test if the vertical movements of blue sharks were linked to behavioural thermoregulation, a time-weighted average of the temperature experienced by sharks was calculated for each integration period, and a non-parametric Mann-Whitney *U* test was performed to determine whether there were significant diel differences in temperature.

## Results

A total of six females, with body-lengths varying from 1.30 to 1.99 m (fork length, FL) were tagged off south-west England. An additional 10 sharks, eight females (0.95 to 2.00 m FL) and two males (1.40 and 2.00 cm FL), were tagged off southern Portugal. Hence, juvenile, sub-adult and adult sharks were tagged in both locations (Table 1, 2). Overall, three tags failed to uplink to Argos satellites. With one exception (tag deployed on shark 1, S1), all reporting tags detached prematurely between 13 and 105 days. Of these, three reported very little archived data and no geolocations, and as a result, no further analyses were performed. Three tags were physically recovered and full archival datasets were downloaded. All analyses were restricted to data recorded prior to premature release dates. Overall, blue shark movements and behaviour were tracked for a total of 401 days, covering an estimated average distance of 1429.40±807.74 km.

**Table 1:** Summary data of the 16 blue sharks tagged with pop-off satellite-linked archival transmitters. F – female; M – male; * no geolocation data received; DNR – did not report.

ID	Fork length (cm)	Sex	Location tagged	Tagging date	Programmed release days	Pop-up location		Pop-up date	Days-at-liberty	Minimum distance (Km)	Minimum distance/day (Km)
Shark 1	199	F	England	06 Jul. 06	30	48.24N	05.99W	05 Aug. 06	30	1058	35.27
Shark 2	153	F	England	21 Jul. 06	60	45.86N	09.52W	10 Aug. 06	20	1403	70.15
Shark 3	130	F	England	08 Aug. 06	60	44.00N	07.91W	29 Aug. 06	21	848	40.38
Shark 4	130	F	England	01 Aug. 07	60	46.38N	10.63W	14 Aug. 07	13	907	69.77
Shark 5	150	F	England	21 Aug. 07	90	46.83N	05.80W	02 Nov. 07	70	2789	39.84
Shark 6	95	F	Portugal	10 Oct. 06	60	34.23N	12.40W	30 Oct. 06	20	614	30.70
Shark 7	115	F	Portugal	04 Oct. 07	60	28.18N	14.70W	15 Nov. 07	42	2029	48.31
Shark 8	200	F	Portugal	03 Jun. 08	120	27.71N	13.96W	15 Aug. 08	73	1232	16.88
Shark 9	180	F	Portugal	04 Jun. 08	120	32.35N	09.67W	04 Aug. 08	61	693	11.36
Shark 10	180	F	Portugal	04 Jun. 08	180	21.99N	27.60W	25 Jul. 08	51	2721	53.34
Shark 11*	110	F	Portugal	12 Oct. 06	120	34.64N	07.42W	28 Oct. 06	16	-	-
Shark 12*	200	F	Portugal	06 Jun. 08	180	47.20N	13.38W	03 Dec. 08	±105	-	-
Shark 13*	200	M	Portugal	11 Jun. 08	180	28.80N	21.16W	22 Jan. 08	±12	-	-
Shark 14	162	F	England	08 Aug. 06	120	DNR					
Shark 15	120	F	Portugal	02 Jun. 08	90	DNR					
Shark 16	140	M	Portugal	09 Jun. 08	120	DNR					

**Table 2:** Summary data for behaviour and environment of tracked female blue sharks.

ID	Fork length (cm)	Life stage	Days-at-liberty	Diel behaviour	Time performed		Water column
					Nr. days	%	
Shark 1	199	Adult	30	Surface oriented	30	100.0	Stratified
Shark 2	153	Sub-adult	20	Surface oriented	20	100.0	Stratified
Shark 3	130	Juvenile	21	Surface oriented	5	23.8	Stratified
				nDVM	16	76.2	Stratified
Shark 4	130	Juvenile	13	rDVM	8	61.5	Isothermal
				nDVM at-depth	3	23.1	Stratified
				nDVM	2	15.4	Stratified
Shark 5	150	Sub-adult	70	rDVM	8	11.4	Isothermal
				nDVM at-depth	22	31.5	Stratified
				nDVM	8	11.4	Frontal
				nDVM at-depth	8	11.4	Stratified
				nDVM	24	34.3	Stratified
Shark 6	95	Juvenile	20	Surface oriented	20	100.0	Stratified
Shark 7	115	Juvenile	42	Irregular	11	26.2	Isothermal
				Surface oriented	31	73.8	Stratified
Shark 8	200	Adult	73	Irregular	73	100.0	Isothermal
Shark 9	180	Adult	61	Surface oriented	20	32.8	Isothermal
				nDVM at-depth	41	67.2	Stratified
Shark 10	180	Adult	51	Irregular	8	15.7	Isothermal
				nDVM	5	9.8	Isothermal
				Irregular	38	74.5	Isothermal

For sharks that shifted between different behaviour types, these are sorted chronologically (top: first; bottom: last).

### Horizontal movements and space-use

**Figure 2:**
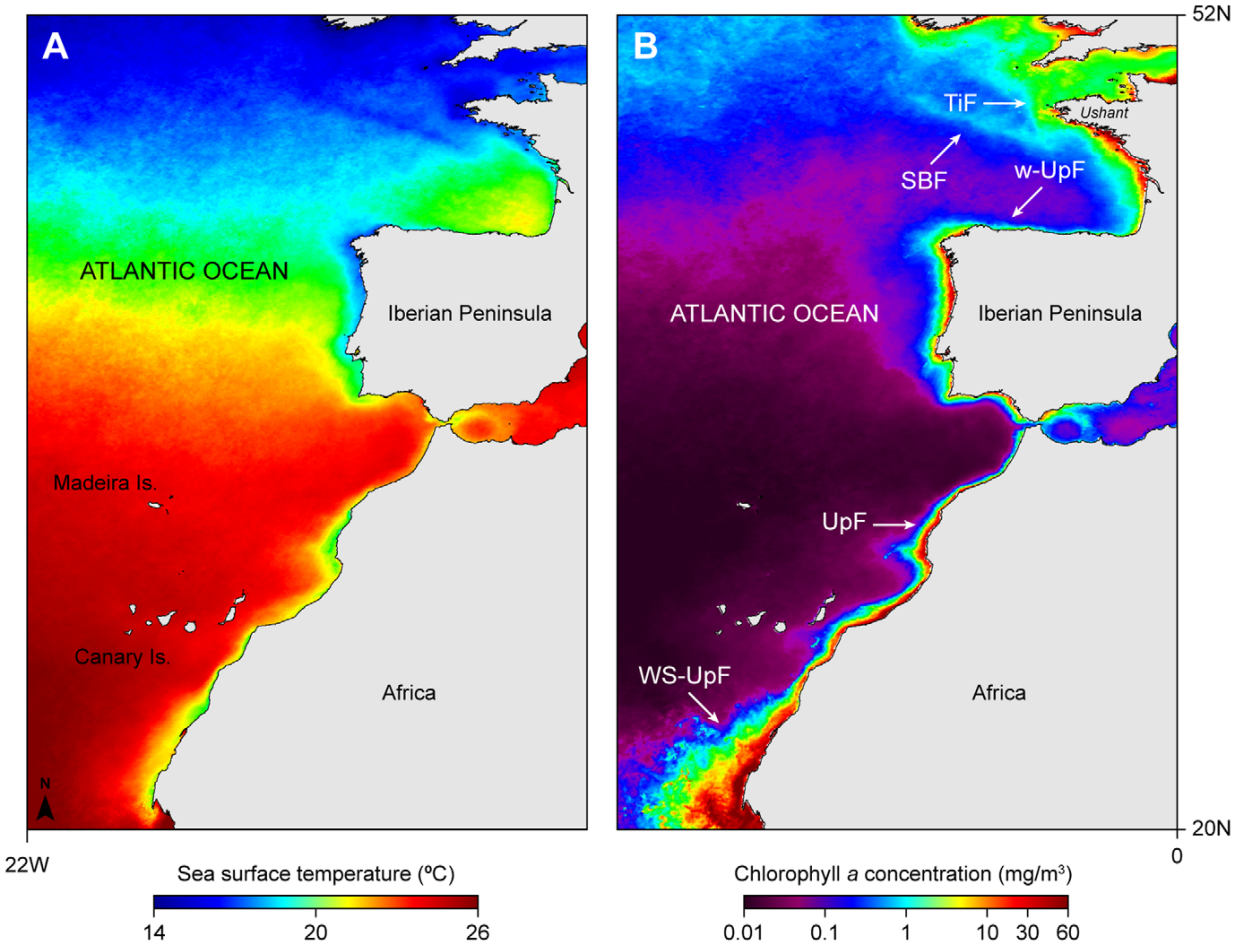
Map of the study area in the North-east Atlantic. Three-year (2006–2008) summer/autumn seasonal average of (A) sea surface temperature and (B) chlorophyll a concentration; TiF, tidal induced front; SBF, shelf-break front; w-UpF, weak upwelling front; UpF, upwelling front; WS-UpF, Western Sahara upwelling front.

Blue sharks generally moved south-west of the tagging sites, both in the English Channel and off southern Portugal (Fig. 1A). Off south-west England, only S1 remained in a restricted area of the continental shelf for the whole duration of the deployment. The general area occupied by S1 was dominated by seasonally persistent tidal fronts, principally the Ushant front (Fig. 2). Shark 2 (S2) moved west into the Celtic Sea shelf area, before moving south into deeper water. Sharks 3 and 4 (S3, S4) moved south-west along the edges of the highly productive Ushant front into the Bay of Biscay region of the continental shelf edge, prior to moving into deeper water; S3 was geolocated in northern Spanish waters in late August. S5 initially moved south, crossing the frontal region, and arrived at the shelf edge in late August, approximately 10 d after tagging. This shark continued to move southeast along the continental shelf edge for ∼18 d, and in late September was geolocated off the northern Spanish coast, where it remained until late October. By early November the shark had moved north and was captured by a longline fishing vessel near the shelf edge. Off southern Portugal, shark 6 (S6) made an initial northward movement into the western coast shelf area before moving south-west into deeper water. Shark 8 (S8) also moved in a south-westerly direction into oligotrophic waters (Fig. 2b) and was geolocated near the Canary Islands in late June, remaining in the area for approximately 50 d. Shark 7 (S7) moved southwest along the African coast, also being geolocated near the Canary Islands by mid-November. Sharks 9 and 10 (S9, S10) were tagged on the same day 1 h apart; both sharks displayed an initial southward movement towards the African coast, with S9 remaining in this upwelling region for ∼40 d, until the tag popped-up in early August. S10 continued to move south, past the Canary Islands, reaching the Western Sahara upwelling system in mid-July (Fig. 1A, Fig. 2).

The estimated space use pattern was statistically different from spatial distributions calculated from random walks (*p*<0.05). The observed high persistence of sharks in some grid cells caused the variance to increase in relation to the average time spent per cell. Shark space use was also correlated with chlorophyll *a* concentration (Spearman Rank correlation; rs = 0.24, p<0.05). Hence, excluding tagging areas (where high space use results from tagging activity itself), spatial activity of tracked female blue sharks concentrated in five major areas (A–E; Fig. 1B). These regions are characterised by the presence of thermal front boundaries and associated enhanced primary production (Fig. 2) [46].

### Diel vertical movements

Blue sharks demonstrated a wide vertical distribution, inhabiting depths from the surface to a maximum of 1160 m (S10), to our knowledge the deepest dive depth recorded for this species, and spanning water temperatures from 7.2 to 27.2°C. Moreover, all sharks displayed significant variations in diving behaviour, phases of which were detected by the split-moving window procedure [3], [45]. Overall, five general behaviour types could be identified, ranging from normal DVM (nDVM) to reverse DVM (rDVM), and including behavioural patterns where no diel differences were apparent.

**Figure 3:**
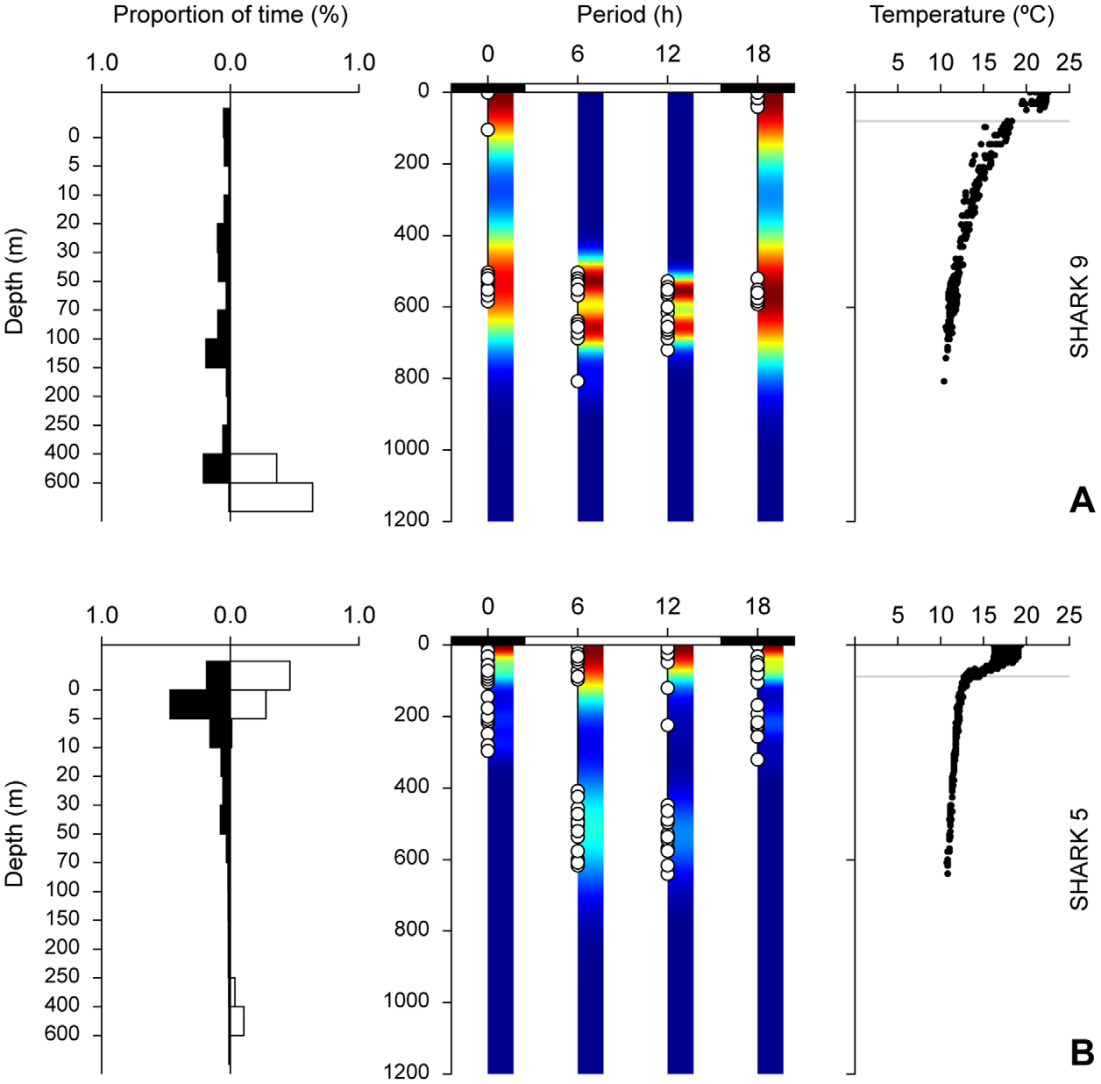
Normal DVM with permanence at depth (A) and normal DVM (B) behaviour plots, from sharks 9 (pooled over 41 days) and 5 (pooled over 24 days), respectively. Left: frequency distributions showing amount of time spent at different depths; black bar denotes essentially night (00:00 and 18:00) and white bar day (06:00 and 12:00). Centre: minimum-maximum depth within each integration interval; white circles represent actual observations; colour represents kernel density estimates for minimum-maximum depth. Right: temperature-at-depth profiles (depth scale is similar to central panel); horizontal grey line represents thermocline depth. Note continued permanence at depth in A.

The first nDVM pattern was characterised by a continued residence at depth during daylight hours, with sharks spending between 70 and 100% of time below the thermocline (∼100 m). Night time time-at-depth (TAD) occupation was irregular, but ∼50% of the time was spent diving in the mixed layer (Fig. 3A). This behaviour was only observed in stratified, productive shelf-edge waters of the Bay of Biscay and off western Africa (Table 2). In the second identified nDVM pattern sharks usually spent ∼80% of the day near the surface, but consistently performed deeper dives during the day than during the night (*p*<0.05, *n* = 346). Daytime maximum dive depths averaged 247±14 m (mean ± SE, n = 177), whereas night time maximum dive depths averaged 180±9 m (*n* = 169). Hence, a second favoured depth range below the thermocline was noticeable, where 10–20% of time was spent. This behaviour was often associated with stratified water (Fig. 3B; Table 2) and the night time depth-range was largely restricted to the mixed layer above the thermocline.

**Figure 4:**
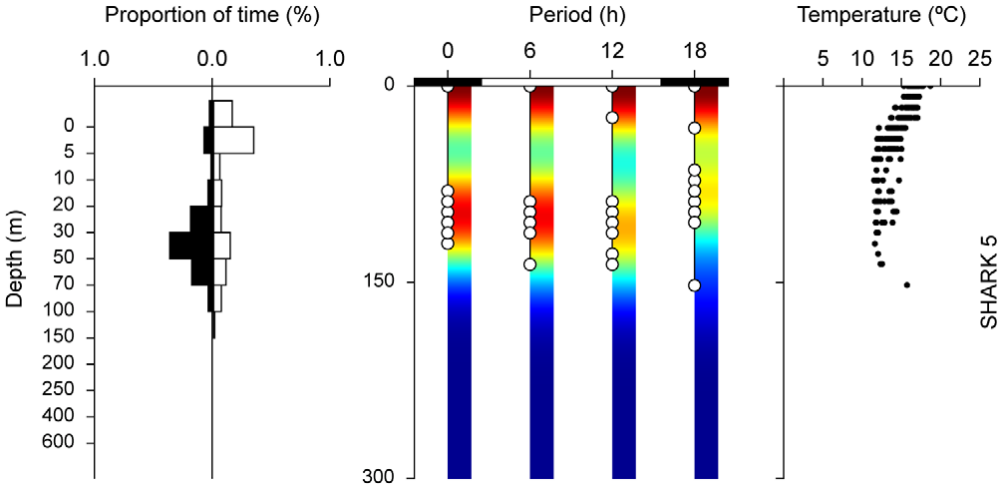
Reverse DVM behaviour plot, from shark 5 (pooled over eight days). Left: frequency distributions showing amount of time spent at different depths; black bar denotes night (00:00 and 18:00) and white bar day (06:00 and 12:00). Centre: minimum-maximum depth within each integration interval; white circles represent actual observations; colour represents kernel density estimates for minimum-maximum depth. Right: temperature-at-depth profiles (depth scale is similar to central panel).

When in shallow shelf waters off England, blue sharks tagged in 2007 displayed a reverse diel vertical migration (rDVM) pattern, spending about 60% of time at depth during the night, and over 60% of time in the top 20 m during the day (Fig. 4). Depth-temperature profiles of the water column indicated sharks were diving through generally isothermal, well-mixed water (Fig. 4, right panel).

**Figure 5:**
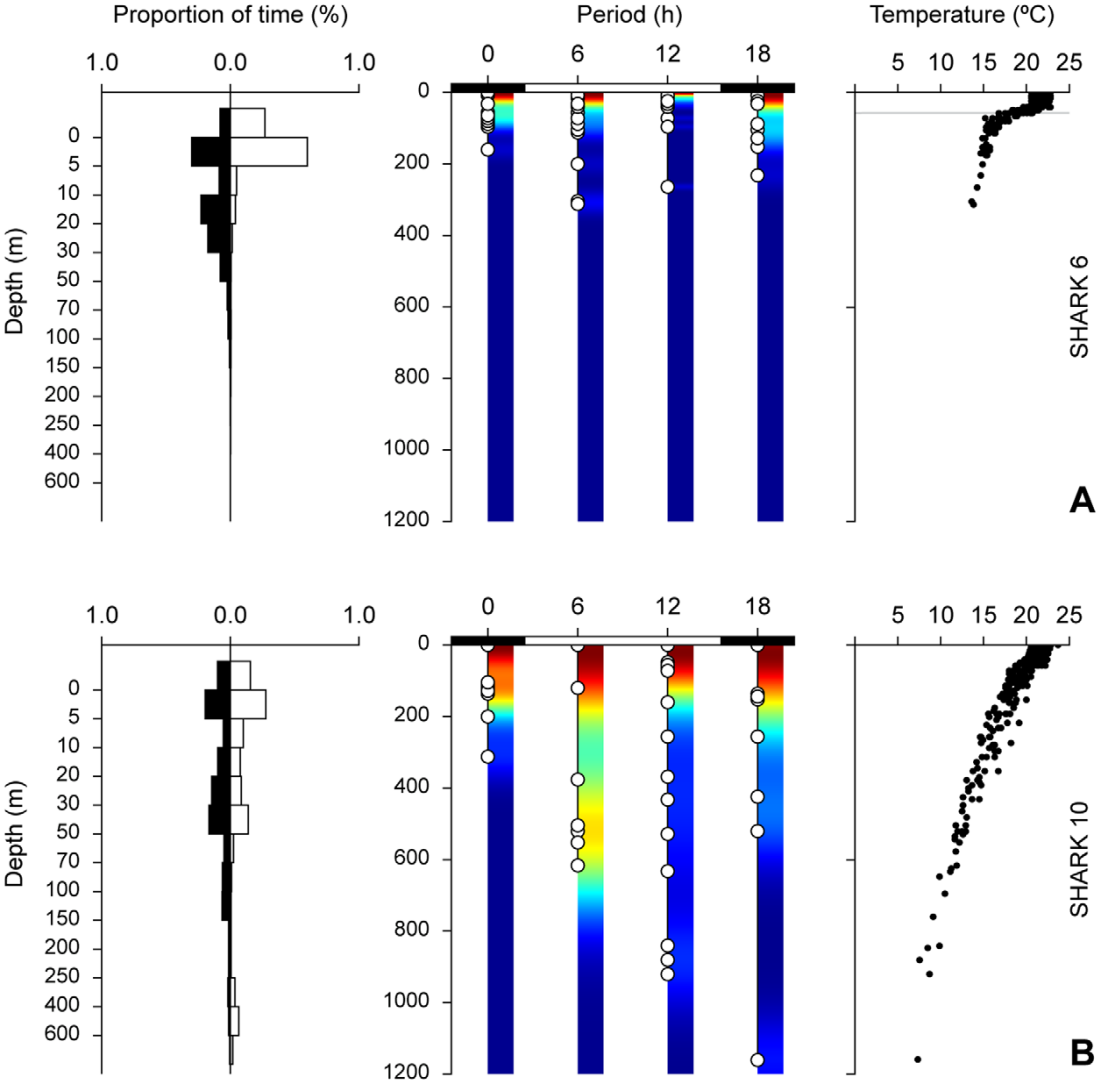
Surface-oriented (A) and irregular (B) diel behaviour plots, from sharks 6 (pooled over 20 days) and 10 (pooled over 38 days), respectively. Left: frequency distributions showing amount of time spent at different depths; black bar denotes essentially night (00:00 and 18:00) and white bar essentially day (06:00 and 12:00) phases. Centre: minimum-maximum depth within each integration interval; white circles represent actual observations; colour represents kernel density estimates for minimum-maximum depth. Right: temperature-at-depth profiles (depth scale is similar to central panel); horizontal grey line represents thermocline depth.

Surface-oriented behaviour was only observed in stratified water with both day and night time TAD occupation generally restricted to thermocline depths (Fig. 5A). Sharks showed no differences between night and daytime behaviour in either TAD or maximum depth (*p* = 0.76, *n* = 253), with individual sharks performing occasional deep, fast dives below the thermocline. Finally, blue sharks in oceanic areas also showed apparently irregular diving behaviour with no obvious differences between light-dark cycles in either TAD or diving depth (*p* = 0.44, *n* = 78), which typically ranged from the surface to ∼200–1160 m (Fig. 5B). Irregular diel behaviour was only displayed in isothermal water (Table 2).

## Discussion

The results of tagging blue sharks with satellite-linked archival transmitters indicate that female sharks display fidelity to localised high-productivity regions characterised by the presence of thermal fronts, at least for the time periods over which they were tracked. Blue shark diel behaviour showed extensive variability, both within and between individuals: diving behaviour ranged from patterns where differences in diel vertical migration were observed, to patterns where no day-night differences were apparent. The study also demonstrated that blue sharks are capable of bathypelagic dives to at least 1160 m.

### Horizontal movements and space-use

Number-tagged blue sharks in south-western English waters and off Ireland have previously been recaptured in the Bay of Biscay in summer and autumn months [37], [38]. Wide-ranging migrations to temperate and warm-water zones have also been described. Although there is no clear seasonal trend in recaptures off West Africa, female blue sharks have been recaptured off the Canary Islands (and further south) in winter and spring months [36], [37], [40]. In this study, blue sharks displayed broad-scale horizontal movements away from the tagging sites, moving across different habitat types, from shelf, shelf-edge and into deep oceanic water. However, movements remaining within spatially restricted areas of the Celtic Shelf, Bay of Biscay and western Africa were significantly different from distributions of model sharks moving according to simple random walks. Blue shark movements were statistically correlated, albeit weakly, with frontal regions. A low correlation between space use and chlorophyll *a* concentration was not unexpected, as there is a spatial and temporal mismatch between shark location and remotely sensed data. The former arises from the limited spatial accuracy of light level geolocation of pop-up satellite tags, whereas the latter derives from the unevenness of remote sensed data (e.g. lack of data as a consequence of cloud cover) which forces pooling at wider temporal scales. Despite these limitations, a general correlation between space use and enhanced productivity areas was present in our tracking data. Our type of study will benefit from the recent development of tags with greater spatial accuracy (e.g. GPS) [47], as well as from advances in remote sensing instrumentation and improvements in interpolation algorithms which have resulted in spatially complete, ultra-high temporal and spatial resolution products.

Frontal zones are typically characterised by high primary and secondary production, where physical processes passively aggregate plankton and therefore generally concentrate higher abundances of prey species [48], [49]. Hence, frontal zones represent regions of forage accumulation [50] and higher *in situ* primary growth [51], and are known to influence the movement and distribution of marine predators [52], [53]. Prolonged residence in productive ‘hotspots’ has been described for several species, from birds [54], [55], fish [17], [20], turtles [56], [57] to marine mammals [58]. In a parallel study in the North-eastern Atlantic, blue sharks tagged with near real-time Argos transmitters, also spent more time in rich frontal zones off the Iberian Peninsula, Bay of Biscay and Celtic Shelf (Fig. S3). Likewise, basking (*Cetorhinus maximus*) and porbeagle sharks (*Lamna nasus*) satellite tracked in the same general area, displayed restricted movements and high space-use of fine to large-scale frontal features [14], [15], [43]. These findings coupled with our results, suggest predators may orientate to high-productivity frontal areas to find food in more predictable habitat then in other areas. Once abundant prey patches are located, predators may remain in these discrete regions for extended periods of time, as seen for tracked blue sharks in this study, which ultimately leads to their spatial aggregation [14]. As well as spending extended periods of time in one area, tracked blue sharks also moved into oligotrophic waters. Although the reasons for these large-scale movements are unknown, they might be linked with dispersing food resources or associated with search for widely distributed prey [58], [59].

### Diel vertical movements

Several hypotheses could be proposed to account for differences in the diel behaviour of pelagic sharks, such as foraging, thermoregulation, predator avoidance and/or orientation. However, as there is no empirical evidence for deep dives (generally performed during daytime) playing a role in the navigational abilities or processes of sharks, diel behavioural orientation seems less likely as an explanation. Of the remaining hypotheses, predator avoidance seems equally unlikely, since we would then expect diel patterns to differ between sharks of different sizes, from juveniles to adults [35]: however, such differences were not observed in satellite tracked sharks in this study (Table 2). If behavioural thermoregulation was responsible for the observed diel patterns the vertical movements of tracked sharks should be regularly linked to thermocline depths, with blue sharks occupying on a daily basis water masses with different temperatures [34]. Again, this hypothesis was not supported by our tracking data (*U* = 63042.5, *t* = 138395.5, *P* = 0.08). Moreover, a recent study of blue sharks in the western Atlantic also demonstrated that diving patterns were not consistent with movements associated with behavioural thermoregulation [60]. Instead, our results indicate that blue sharks belonging to varying age groups exhibited similar diel behavioural strategies at different geographical areas, thus supporting the hypothesis that differences in diel behaviour were likely linked to foraging.

In well-stratified off-shelf regions, zooplankton organisms predominantly undertake regular vertical movements, generally occurring at depth during the day and approaching the surface during the night. Downward migrations of zooplankton occur at sunrise and upwards at sunset and are thought to be an adaptive response to reduce predation risk [23]. Normal DVM diving patterns were commonly observed in blue sharks tracked by us, but there were significant differences in diel diving behaviour. In shelf-break regions blue sharks displayed a continuous residence at depth during the day, while swimming near the surface at night. This pronounced diel migratory pattern is consistent with tracking vertically migrating prey, such as squid, suggesting sharks maximised the time spent within a prey patch (see below). This behaviour has recently been observed for blue sharks in the northwest Atlantic [60], suggesting that residence at depth may be a more common occurrence than previously thought in frontal systems, such as the Gulf Stream or the Biscay shelf edge. Thus far, such marked nDVM behaviour has only been observed in relatively few large predatory fish, such as bigeye tuna (*Thunnus obesus*) [33], swordfish (*Xiphias gladius*) [61] and bigeye thresher shark (*Alopias superciliosus*) [62].

In this study, blue sharks displaying the second nDVM pattern spent more time near the surface during the day and night, but displayed a distinct normal diel component in maximum depth, repeatedly diving deeper during the day, while night time excursions were frequently limited to thermocline depths. Deep descents below the mixed layer during daylight hours most likely represent foraging dives. Furthermore, the uniformity of dive depth (between 200–400 m) implies prey patches remained in a confined depth layer. The stomach content of blue sharks also suggests foraging at depth, with deep-water squid known to be an important item in the diet of this species in the region where we tracked them [63]. An ongoing study in the North-eastern Atlantic reveals a clear dominance of cephalopod prey items in the stomach contents of longline-caught blue sharks (index of relative importance, IRI, of 94%); deep-water squid species such as *Vampyroteuthis infernalis* and *Mastigoteuthis* sp. were commonly identified (N. Queiroz, unpublished data). This indicates blue sharks may forage on cephalopods at considerable depths.

Over the European continental shelf, blue sharks in well-mixed or weakly stratified water showed a pattern consistent with reverse diel vertical migration (rDVM) behaviour. A similar behavioural pattern has been described for basking and porbeagle sharks tracked over the continental shelf [17], [43]. Interestingly, rDVM behaviour was observed in basking sharks foraging in inner-shelf regions characterised by tidal fronts [17]. In the former study, reverse DVM of tracked sharks was linked to zooplankton behaviour and distribution, which aggregated in surface waters during daytime, possibly influencing schooling fish distribution. Hence, a possible reason for the rDVM seen in tagged blue sharks here was tracking of pelagic fish (e.g. mackerel, *Scomber scombrus*), or of the predators of small pelagic fish such as squid.

Billfish and tuna species generally exhibit a marked preference for the mixed layer, rarely crossing the thermocline, e.g. [64], [65]. Likewise, blue sharks tracked in this study displayed a similar surface-oriented behavioural pattern in stratified oceanic regions, where no apparent diel differences in diving were observed. Surface-oriented behaviour could indicate near-surface prey patches are dense enough for high encounter rates [2]. Finally, irregular diving seen in tracked blue sharks was probably related, directly or indirectly, to changes in the thermal structure of the water column [45]. Strikingly, this behavioural pattern was observed in S8 and S10 while the sharks were associated with well-mixed waters, typical of the Western Sahara upwelling region [66]. Overall, shifts in the diel behaviour of blue sharks may be related to changes in prey type or density, e.g. low prey levels near the surface may induce foraging at depth [2], although in some circumstances, these behavioural changes may have also been direct responses to the thermal profile of the water column, which can influence the availability of prey resources [17].

Future research in this area would benefit from studies focusing on resolving the movements of predatory sharks, and other large pelagic vertebrates, in relation to the distribution and abundance of their prey species. This would enable more process-based investigations aimed at identifying how and under what conditions sharks search for prey. Furthermore, there is a need for more information about the movements of male sharks, and for both sexes in the winter and spring period so as to build a more complete picture of blue shark population structuring in the eastern north Atlantic [67], which will be important for their conservation and management in the face of high fishing pressure.

### Fisheries and behaviour

**Figure 6:**
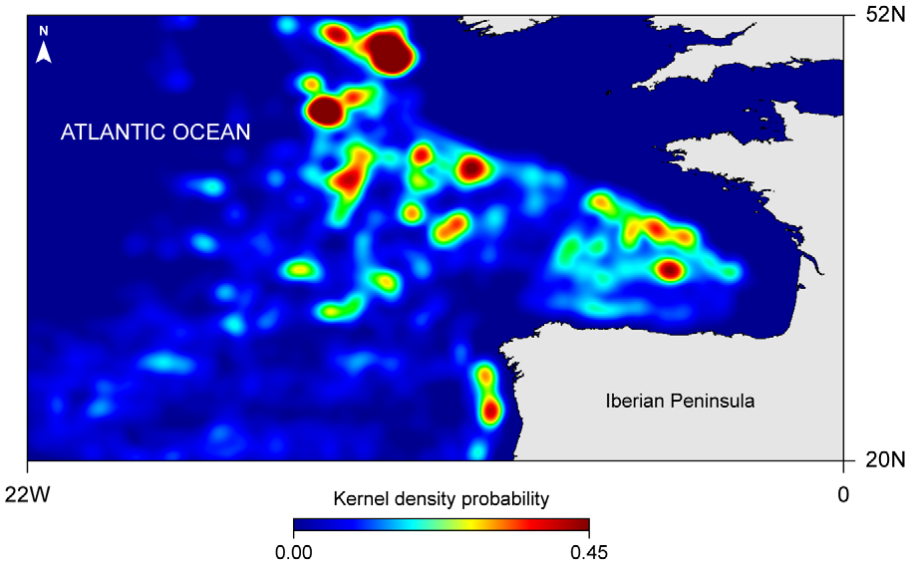
Kernel density estimation plot of 3-year (2006–2008) summer/autumn longline space-use in the northeast Atlantic derived using vessel monitoring system (VMS) data.

Blue sharks are commonly caught as bycatch in longlines targeting swordfish and tuna species, e.g. [10]. Pelagic longlines comprise a mainline, which can extend up to 100 km in length, suspended in the water column, with baited hooks on branch-lines attached at evenly spaced intervals. Longlines are generally deployed overnight and hooks set at depths typically ranging from ∼100 to 300 m [22], [68]. Given that the observed blue sharks night-time habitat was generally restricted to near-surface depths (i.e. surface to ∼100 m), there is therefore a spatial and temporal overlap between the longline fishing effort and the vertical niche of blue sharks. Hence, analysis of the recorded tracks suggest the vertical overlap between blue shark night-time occupation in open ocean areas and hook depth, ranges from ∼76% to as high as 100%. Strikingly, VMS data of pelagic longliners operating in the northeast Atlantic shows longlining activities significantly concentrate in high productivity areas (r_s_ = 0.56, *p*<0.05; Fig. 6). Space-use patterns of satellite-linked sharks suggest they spend much of the time in the same type of areas where longline fisheries target commercially important marine fish, which could account for the high levels of by-catch of blue sharks and the rapid declines reported for this species [6], [12]. Consequently, high productivity regions may be ideal habitat targets for the implementation of high seas marine protected areas (MPAs), not only for blue sharks, but also for other large pelagic predators.

## Supporting Information

Figure S1 **Longline yearly averaged effort data by 5×5 degree squares for the North Atlantic (1972–2003) and Pacific (1950–2004); class breaks were determined statistically by finding adjacent feature pairs between which there was a relatively large difference in data value – natural breaks.**
(TIF)

Figure S2 **Density grid of number of hours spent per 0.5×0.5° unit area for observed (A) and three different simulated particles (B–D).**
(TIF)

Figure S3 **Movement and high space use areas occupied by smart position-only transmitting (SPOT) tagged blue sharks.** (A) General movement patterns overlaid on bathymetry; black circles denote last transmission locations and white circles geolocated positions. (B) Kernel density plot of 3-year (2006–2008) summer/autumn seasonal average of (C) sea surface temperature and (D) chlorophyll *a* concentration. Note prolonged residence off the Iberian Peninsula wind-driven upwelling region, shelf-break and tidal induced fronts in the Bay of Biscay and Celtic Sea, respectively (Spearman Rank correlation; r_s_ = 0.45, p<0.05).
(TIF)
