# Supplementary figures and images for: Spatial Dynamics and Expanded Vertical Niche of Blue Sharks in Oceanographic Fronts Reveal Habitat Targets for Conservation
Source: PLoS One. 2012 Feb 29;7(2):e32374. doi: 10.1371/journal.pone.0032374 (PMC3290575; doi:10.1371/journal.pone.0032374)

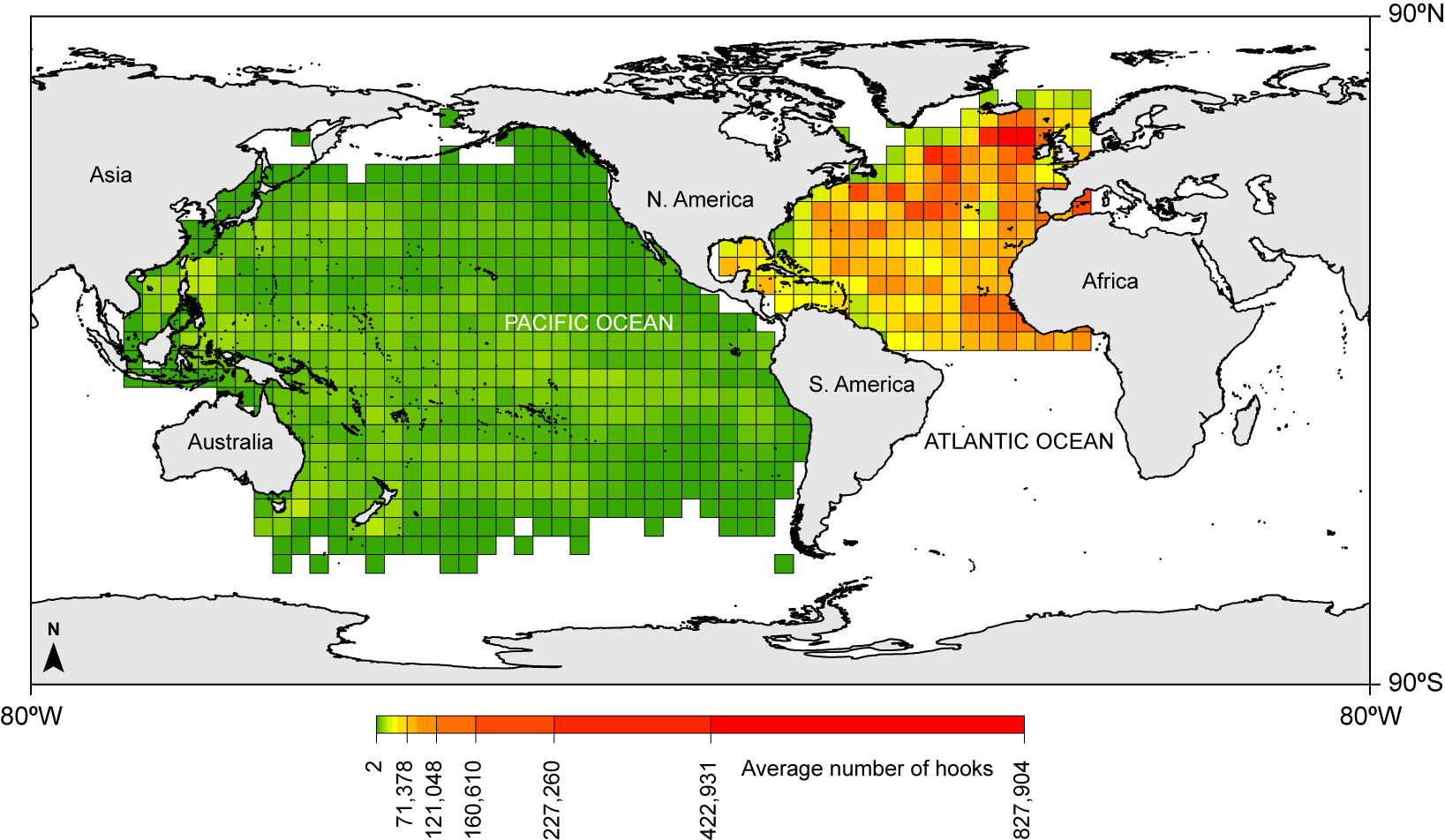

Supplement: Figure S1 — Longline yearly averaged effort data by 5×5 degree squares for the North Atlantic (1972–2003) and Pacific (1950–2004); class breaks were determined statistically by finding adjacent feature pairs between which there was a relatively large difference in data value – natural breaks. (TIF) [file pone.0032374.s001.tif]

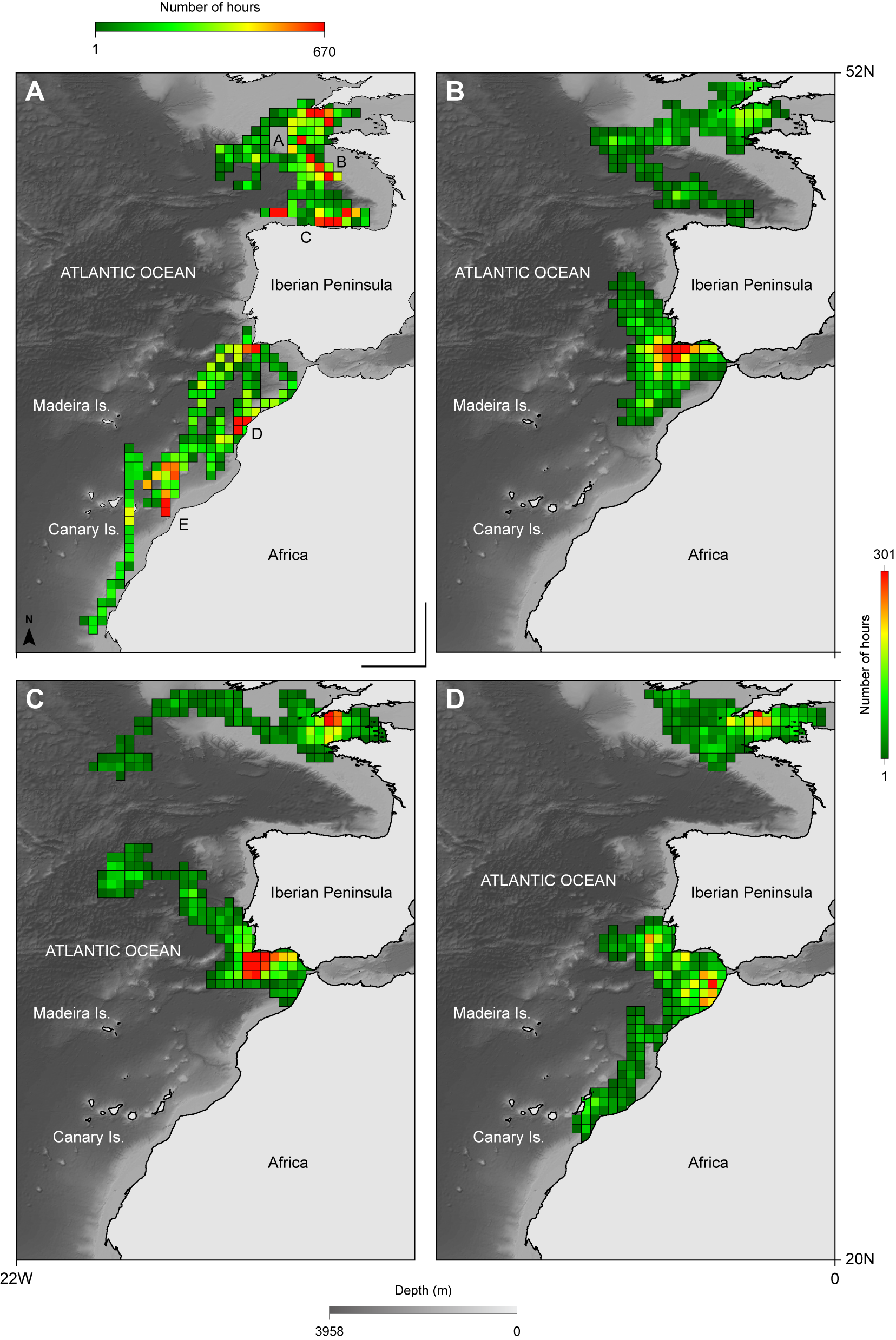

Supplement: Figure S2 — Density grid of number of hours spent per 0.5×0.5° unit area for observed (A) and three different simulated particles (B–D). (TIF) [file pone.0032374.s002.tif]

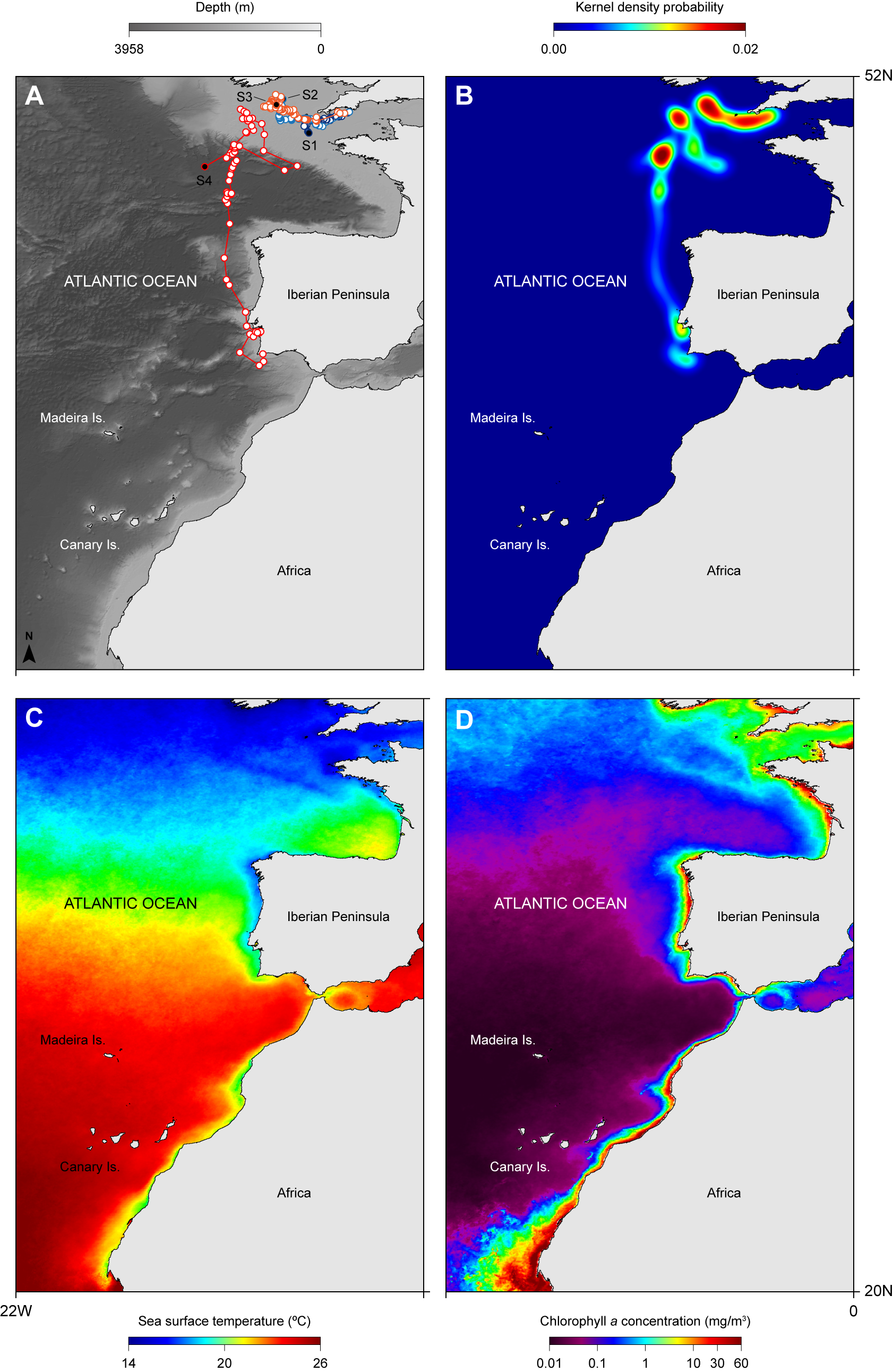

Supplement: Figure S3 — Movement and high space use areas occupied by smart position-only transmitting (SPOT) tagged blue sharks. (A) General movement patterns overlaid on bathymetry; black circles denote last transmission locations and white circles geolocated positions. (B) Kernel density plot of 3-year (2006–2008) summer/autumn seasonal average of (C) sea surface temperature and (D) chlorophyll a concentration. Note prolonged residence off the Iberian Peninsula wind-driven upwelling region, shelf-break and tidal induced fronts in the Bay of Biscay and Celtic Sea, respectively (Spearman Rank correlation; rs = 0.45, p<0.05). (TIF) [file pone.0032374.s003.tif]
